# Global practices in adapting cognitive behavioral therapy for ethnoracial populations with substance use disorders: a scoping review of adaptation strategies, treatment outcomes, and geographic gaps

**DOI:** 10.1186/s13033-026-00730-z

**Published:** 2026-09-03

**Authors:** Obi Onyegesi, Patrick M. W. Kennedy, Skyler Hamilton, Lesia M. Ruglass, Fiona N. Conway

**Affiliations:** 1 School of Social Work, The University of Texas at Austin, 405 W 25th St, Austin, 78705 TX USA; 2 Addiction Research Institute, School of Social Work, The University of Texas at Austin, Austin, TX USA; 3 Department of Psychology, The City College of New York, New York, NY USA

**Keywords:** Cognitive behavioral therapy, Cultural adaptation, Ethnoracial adaptation, Implementation science, Scoping review, Substance use disorders

## Abstract

**Background:**

Cognitive behavioral therapy (CBT) is an evidence-based treatment for substance use disorders (SUDs), yet standard protocols may have limited cultural relevance for ethnoracially diverse populations. Cultural adaptation may enhance the fit and relevance of interventions; however, the scope, characteristics, and outcomes of ethnoracial adaptations of CBT for SUD remain underexamined. This scoping review mapped the global evidence on ethnoracially adapted CBT for SUDs, including strategies, implementation characteristics, and treatment outcomes.

**Methods:**

Following PRISMA-ScR guidelines, we systematically searched nine databases for peer-reviewed empirical studies evaluating ethnoracially adapted CBT interventions for SUDs. Studies were included if they explicitly described an ethnoracial adaptation to CBT. Extracted data included study design, population characteristics, strategies, comparator conditions, implementation indicators, and substance use outcomes.

**Results:**

Of 671 records identified, 12 studies met the inclusion criteria, representing populations in the United States and Kenya. Most studies focused on Latino/Hispanic populations living in the United States, with comparatively limited representation of other ethnoracial groups and low- and middle-income country contexts. Adaptations included both surface-structure modifications (e.g., language translation, culturally relevant examples, and tailored delivery formats) and deep-structure modifications that incorporated culturally relevant values, belief systems, and lived experiences into core CBT content and delivery. Most studies reported favorable substance use outcomes following participation in ethnoracially adapted CBT interventions. Several studies also identified culturally salient factors, including ethnic identity, *familismo*, and religiosity, as moderators or correlates of treatment response. However, implementation outcomes such as feasibility, fidelity, and acceptability were inconsistently reported, long-term follow-up data were limited, and only a small number of studies directly compared ethnoracially adapted CBT with standard CBT.

**Conclusions:**

The current literature suggests that ethnoracially adapted CBT is a promising approach for addressing SUDs among diverse populations. However, limitations in implementation reporting, long-term outcome assessment, geographic representation, and direct comparisons with standard CBT constrain conclusions regarding the specific contribution of cultural adaptation to treatment outcomes. Future research should expand representation across ethnoracial groups and geographic regions and clarify how cultural adaptations influence treatment engagement, implementation, and substance use outcomes.

## Background

Substance use disorder (SUD), defined as persistent substance use contributing to clinically significant distress or impairment [1], remains a pressing global public health concern [2]. An estimated 250 million people aged 15–64 years report drug use, with over 35 million meeting criteria for SUD [3]. More concerningly, SUDs contribute to 11.8 million premature deaths annually and account for more than 5% of the global burden of disease, as measured by disability-adjusted life years (DALYs), which capture both years lost to early death and years lived with disability [4–8]. This burden is expected to grow disproportionately in low- and middle-income countries (LMICs). By 2050, nearly 70% of the world’s population will reside in Asia or sub-Saharan Africa, where the SUD burden is projected to increase by 130%, resulting in an estimated 45 million DALYs [9–11]. Yet, despite the magnitude of this crisis, treatment access remains alarmingly limited: globally, roughly only one in eight individuals with SUD receives any form of care [3, 12]. Compounding this inequity, most empirical evaluations of evidence-based SUD treatments are conducted in high-income countries, while LMICs remain significantly underrepresented in the research literature, even as the burden is rising most rapidly there. Although precise data are scarce, recent reviews suggest that only a small proportion of SUD intervention trials are conducted in LMICs, revealing a critical gap between where the need is greatest and where evidence is being generated [13–15].

Cognitive-behavioral therapy (CBT) is one of the most empirically supported interventions for SUDs [16, 17] Rooted in social learning, cognitive, and behavioral theories, CBT conceptualizes substance use as a learned behavior reinforced by environmental and social cues [18–21]. It focuses on developing awareness of triggers, restructuring maladaptive beliefs, and teaching coping skills [22, 23]. CBT has demonstrated efficacy across a range of substances (e.g., alcohol, stimulants), delivery formats (e.g., individual, group, web-based, digital), and treatment settings (e.g., inpatient, outpatient) [18, 24–27]. It is used in 94% of U.S. treatment programs in the United States (U.S.) [28]. Globally, it is endorsed by major health institutions [26, 29, 30]. However, CBT’s evidence base is predominantly derived from studies with White, Western, and middle-class participants [18, 31, 32]. This raises concerns about its cultural generalizability, particularly given that CBT’s core assumptions, such as individualism, rational thinking, and autonomous change, may not align with collectivist or relational worldviews common in many non-Western LMICs [33]. Furthermore, stigma and cultural perceptions regarding SUD and its treatment can influence access, engagement, and outcomes [34–38].

Cultural adaptation refers to the intentional modification of an evidence-based intervention to enhance its relevance, acceptability, and effectiveness across cultural groups, while honoring their cultural knowledge, values, and norms [30, 39–43]. Ethnoracial cultural adaptation (or ethnoracial adaptation) extends this process to interventions designed for specific ethnoracial groups, defined as individuals who self-identify as sharing sufficient racial and/or ethnic characteristics to be recognized as members of a particular group [44]. Adaptations range from surface-structure (e.g., bilingual materials, culturally relevant imagery, and the incorporation of familiar lifestyle elements such as music or food) to deep-structure changes that revise core components of the intervention to better align with a group’s lived experience and protective factors [45, 46]. Notably, several studies emphasized that cultural adaptation is most effective when developed through community engagement, with participants and stakeholders serving not only as recipients of care but also as co-designers of intervention content, format, and delivery [47–50]. Such participatory approaches strengthen the cultural validity, acceptability, and sustainability of CBT models [47–50]. When thoughtfully implemented, culturally adapted interventions can also enhance treatment engagement, retention, and outcomes [51–57].

Despite the recognized importance of these adaptations, there remains a significant gap in the literature on the surface-to-deep structure ethnoracial adaptations of CBT for SUD. Windsor and colleagues conducted a meta-analysis comparing CBT substance use outcomes across studies with predominantly non-Hispanic White samples and studies with predominantly Black and/or Hispanic samples [58]. Although CBT was associated with reductions in substance use across groups, pre-post treatment effect sizes were significantly larger in predominantly non-Hispanic White studies than in predominantly Black/Hispanic studies. However, no significant racial differences were observed when CBT was compared with alternative treatment conditions. Because none of the included studies reported outcomes separately for Black or Hispanic participants and all samples included White participants, the authors cautioned that these findings should be considered preliminary [59]. Similarly, Venner and colleagues conducted a scoping review of cultural adaptations of SUD treatments for Latinx communities [42]. The review found that culturally adapted evidence-based treatments, including CBT-based interventions, generally produced significant improvements in substance use outcomes over time, and several studies reported superior outcomes relative to standard interventions or comparison conditions. The review also identified cultural and social factors as significant moderators of treatment response. These include *familismo* (a collectivist value emphasizing family warmth, closeness, and prioritization of family over self), experiences of discrimination, and ethnic identity (an individual’s self-concept shaped by nationality, ethnicity, religion, and language) [60–62]. The review indicated that individuals reporting stronger cultural identity, higher parental familism, or greater baseline discrimination often demonstrated greater benefit from culturally adapted interventions. However, both studies focused primarily on studies conducted in the United States, leaving limited evidence regarding the effectiveness of culturally adapted CBT interventions for SUDs in ethnoracial populations outside the U.S. context [58, 63]. Consequently, the extent to which ethnoracial adaptations of CBT are being implemented and evaluated across diverse cultural and geographic settings remains unclear.

## Purpose of the study

This scoping review systematically maps the global evidence on ethnoracial adaptations of CBT for SUDs. Unlike systematic reviews, which typically focus narrowly on intervention efficacy, scoping reviews are particularly useful for clarifying how key concepts related to cultural adaptation are defined, operationalized, and examined across diverse populations and settings [64, 65]. This approach is well-suited to fragmented or emerging fields, as it facilitates the identification of conceptual, methodological, and implementation-related gaps that can guide future research and practice.

By mapping the global evidence on ethnoracially adapted CBT for SUDs, this review aims to inform the development, implementation, and dissemination of culturally responsive, evidence-based interventions within mental health and substance use treatment systems worldwide. Specifically, the goals of this review are to:

1. Comprehensively map the global literature on ethnoracial adaptations of CBT for SUDs, including studies conducted in both high-income countries and LMICs;
2. Describe the surface- and deep-structure adaptation strategies used to enhance ethnoracial relevance and responsiveness; and
3. Examine reported treatment outcomes and implementation characteristics of ethnoracially adapted CBT interventions.

To maintain conceptual and cultural fidelity, we reported cultural constructs, adaptation processes, target populations, and outcomes using the terminology employed by the original study authors. By synthesizing evidence across diverse cultural and geographic contexts, this review seeks to strengthen understanding of how ethnoracial adaptations are incorporated into CBT for SUDs and to inform future efforts to expand equitable, culturally responsive, and evidence-based care globally.

## Methods

### Search strategy

This scoping review focused on empirical studies examining ethnoracially adapted CBT interventions for SUDs. We employed the Mak & Thomas methodology for scoping reviews and registered our review on the Open Science Framework (registration DOI:https://doi.org/10.17605/OSF.IO/9PBCR) [66]. In collaboration with a university librarian, we developed a search strategy to identify all relevant published studies. The initial search was conducted in February 2025, with an updated search in May 2026. Substance use terms included general descriptors: substance use OR substance abuse OR drug use OR drug abuse OR dependence OR addiction OR alcoholism OR alcohol dependence OR alcohol abuse OR alcoholic OR alcohol addiction. The terms “cognitive behavio* therapy” OR “CBT” OR “cognitive therapy” OR (behavio* n1(therapy OR treatment OR intervention)) OR “talk* therapy” OR “counsel*”)” and cultur* n1 (adapt* OR relevant OR responsive OR sustaining)” were utilized to identify articles pertinent to the selected intervention modality (i.e. cognitive-behavioral therapy) in nine major databases: APA PsycInfo, CINAHL, Education Source, Health Source, ERIC, Psychology and Behavioral Sciences Collection, PubMed, Science and Technology Collection, and SocIndex. We included all peer-reviewed journal articles written in English published prior to May 2026. We also reviewed the reference lists from prior systematic reviews on CBT interventions for SUDs to identify additional studies that met our inclusion criteria.

### Inclusion and exclusion criteria

First, we included studies based on CBT or that incorporated CBT as a central component. These studies employed individual, group, digital, or integrated delivery formats. Second, CBT interventions were required to include an explicit ethnoracial adaptation, that is, deliberate modifications to the content, delivery, or implementation of CBT intended to enhance its cultural relevance, acceptability, and appropriateness for a specific ethnoracial group [30, 33, 40, 44]. Finally, only empirical, peer-reviewed studies published in English that used qualitative, quantitative, or mixed methods designs (e.g., RCTs, quasi-experimental studies, case studies, pilot or implementation research) were included.

We excluded studies that did not meet these criteria. Specifically, we excluded theoretical or conceptual papers, review articles, opinion pieces, conference abstracts, dissertations, and other non-peer-reviewed materials. We also excluded studies describing standard CBT protocols without explicit ethnoracial cultural adaptation, as well as studies in which adaptations were developed solely for non-ethnoracial populations (e.g., gender-specific, veteran-specific, or occupation-specific adaptations). Studies that did not identify substance use or substance use disorder as a treatment target were also excluded.

### Screening approach

All articles identified through the search were imported into the Rayyan software for management and de-duplication. Screening was conducted in a multi-step process by two independent reviewers (PK and SH). First, titles and abstracts were reviewed to assess initial eligibility. Full-text screening was conducted for articles that met the inclusion criteria at the title/abstract stage. Discrepancies between reviewers were resolved through discussion and, when necessary, adjudicated by a third reviewer (OO).

### Data extraction

The following data were extracted from each study: (1) lead author and year of publication; (2) research design; (3) information regarding shared samples or related trials, when applicable; (4) sample characteristics, including target population, age, sample size, race/ethnicity, and sex/gender; (5) intervention setting; (6) primary substances targeted; (7) ethnoracially adapted CBT-specific components; (8) control or comparison conditions, when applicable; (9) outcome measures; (10) key findings; and (11) country of implementation. Extracted data were organized in a structured Excel spreadsheet and summarized using descriptive counts for study characteristics and narrative synthesis to identify patterns in cultural adaptations, outcomes, and implementation factors. Reporting of this review followed the Preferred Reporting Items for Systematic Reviews and Meta-Analyses Extension for Scoping Reviews (PRISMA-ScR) [67].

## Results

### Included studies

**Fig. 1:**
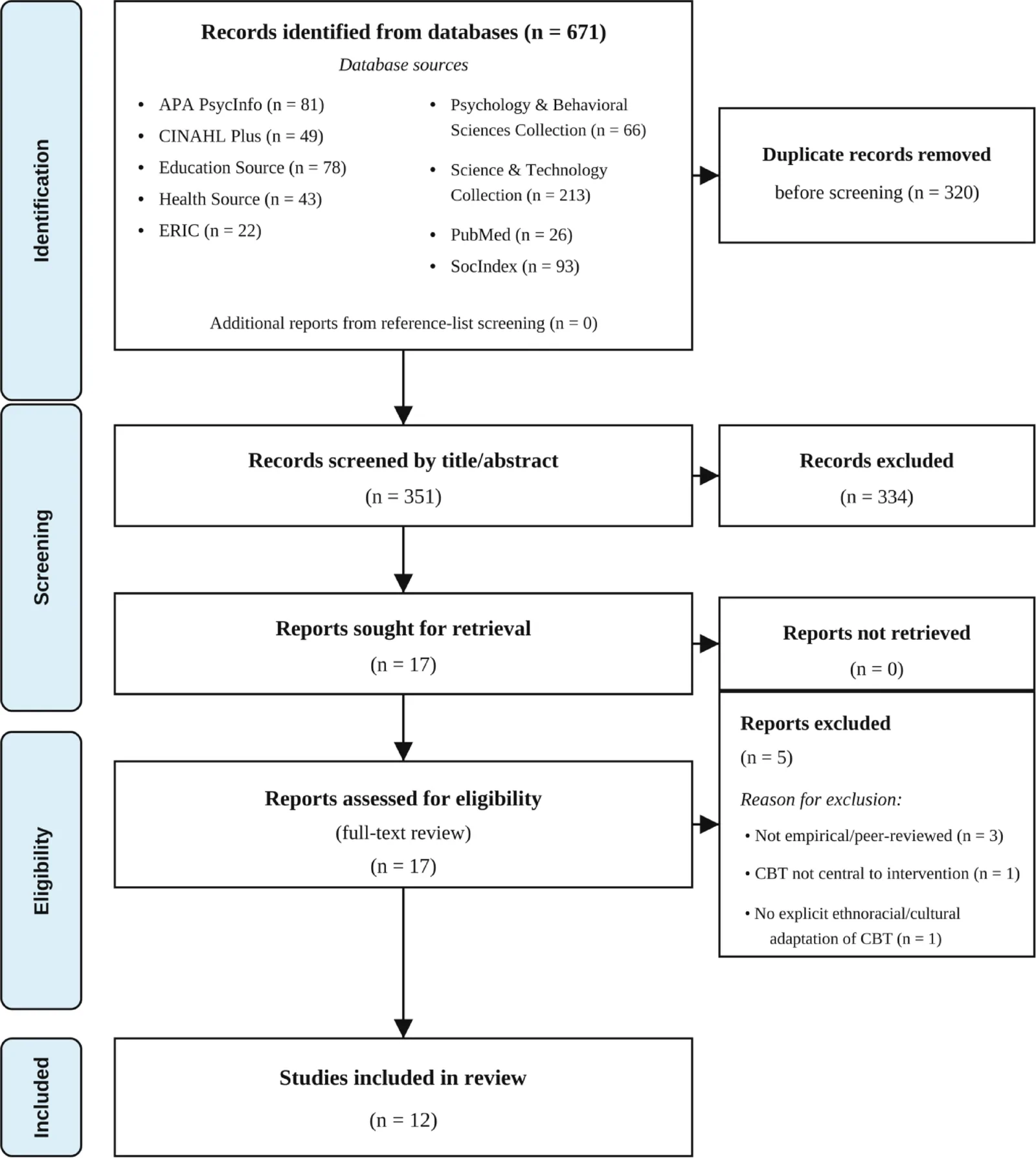
PRISMA Flow Diagram. Flow diagram illustrating the identification, screening, eligibility assessment, and inclusion of studies for the scoping review following PRISMA-ScR guidelines

The database search yielded 671 records, resulting in 351 unique titles after deduplication. Following a full-text review, 12 studies met the inclusion criteria. Table 1 below summarizes the characteristics of the included studies. Figure 1 (PRISMA Flow Diagram) illustrates the selection process.

**Table 1:** Studies of culturally adapted cognitive-behavioral therapy interventions for substance use disorder (*N* = 12)

Author(s), Year	Research Design	Shared Sample/Related Trial	Target Population (Age)	Size (*N*)	Race/Ethnicity	Sex/Gender	Setting	Primary Substance	CBT-Specific Component Adapted	Control Condition	Outcome Measures	Outcomes	Country
***Benitez et al.***,*** 2025***	Secondary analysis of a randomized control trial (RCT)	Secondary analysis of Paris et al., 2018 RCT (same sample) (Clinical Trial ID: NCT02043210)	Hispanic adults with substance use disorder (SUD) (Mean age [SD], years = 43 [11])	85	Hispanic	Male- 68%, Female- 32%	Outpatient clinics	Cannabis, alcohol, cocaine	Language; cultural themes and values	Treatment as usual (TAU)	Percentage of days abstinent (PDA) from substances; session attendance; treatment satisfaction; Spanish DRRT for coping skills quality; daily substance use in the past 28 days (Timeline Followback [TLFB]); urine/breathalyzer verification	Treatment-exposed participants receiving TAU + Spanish CBT4CBT demonstrated greater improvement in quality of coping skills during treatment compared to TAU alone. This difference was not detected during the 3-month follow-up period. Across the full sample and treatment-exposed subsample, participants with higher quality coping skills reported lower substance use	USA
***Burrow-Sánchez & Wrona***,*** 2012***	Pilot RCT	Pilot study preceding Burrow-Sánchez et al., 2015 RCT; (Clinical Trial ID not reported)	Latino adolescents with SUD (Mean age [SD], years = 15.49 [1.34])	35	Latino	Male- 94%, Female- 6%	Outpatient SUD treatment programs; primarily juvenile justice referrals	Alcohol and any illicit drugs	Language; cultural themes and values; inclusion of Ethnic Identity and Adjustment module; ethnic identity matching; increasing family involvement	S-CBT compared with A-CBT	Number of days a substance was used in the past 90 days; treatment retention; treatment satisfaction	Both S-CBT and A-CBT groups demonstrated significant reductions in substance use from pre- to post-treatment, with slight increases at 3-month follow-up. Higher ethnic identity and familism moderated treatment effects, with adolescents in the A-CBT condition reporting the lowest substance use levels. Retention and satisfaction were high across conditions	USA
***Burrow-Sánchez et al.***,*** 2015***	RCT	Follow-up RCT to Burrow-Sánchez & Wrona, 2012 pilot RCT (Clinical Trial ID not reported)	Latino adolescents with SUD (Mean Age [SD], years = 15.2 [1.24])	70	Latino/Hispanic	Male- 90%, Female- 10%	Group-based substance abuse treatment; primarily juvenile justice referrals	Alcohol, marijuana, and polysubstance use	Ethnic identity and adjustment module; culturally relevant examples and role-plays; discussion of acculturation and discrimination; increased bilingual parent contact/family involvement	Standard manualized CBT (S-CBT) compared with culturally accommodated CBT (A-CBT)	Number of days a substance was used in the past 90 days (TLFB)	Both S-CBT and A-CBT conditions produced significant decreases in substance use over time, but there was no significant time × treatment interaction. Adolescents in the A-CBT condition with higher ethnic identity/familism generally reported fewer days of substance use	USA
***Burrow-Sánchez et al.***,*** 2019***	RCT	Follow-up analysis of Burrow-Sánchez et al., 2015 RCT (same sample; no separate trial registration reported)	Latino adolescents with SUD (Mean age = 15.2 years [1.24])	70	Latino/Hispanic	Male − 90% Female − 10%	Community-based group substance abuse treatment; majority referred through the juvenile justice system	Alcohol, marijuana, polysubstance use	Inclusion Ethnic Identity and Adjustment module; culturally relevant role-plays and examples; discussion of discrimination, racism, and acculturation-related stressors; increased parent contact and family involvement	Standard manualized CBT (S-CBT) compared with culturally accommodated CBT (A-CBT)	TLFB (days of substance use in past 90 days); Acculturation Rating Scale for Mexican Americans-II (ARSMA-II); MEIM; Familism Scale	Adolescents receiving A-CBT reported significantly lower substance use at the 12-month follow-up compared with S-CBT. Parental familism moderated treatment outcomes, with adolescents in the culturally accommodated condition reporting lower substance use than their S-CBT counterparts across both low- and high-familism subgroups	USA
***Jaramillo et al.***,*** 2022***	Secondary analysis of RCT	Secondary analysis of Paris et al., 2018 RCT (Clinical trial ID: NCT02043210)	Primary Spanish-speaking Latinx adults with SUD (Mean Age [SD], years = 43.28 [11.4])	89	Latinx- 83%, White- 7.6%, Native American- 2.2%, Black/African American- 1.1%, Did not answer- 3.3%	Male- 70%, Female- 30%	Outpatient substance use treatment clinics	Alcohol, cannabis, cocaine, opioids/stimulants	Spanish-language delivery; inclusion of culturally relevant storylines and Latinx characters; themes of immigration and intergenerational conflict; inclusion of cultural values (e.g., *familismo*,* machismo*,* respeto*,* personalismo*); use of telenovela-style format	TAU compared with TAU plus culturally adapted Spanish-language CBT4CBT	PDA; maximum consecutive days abstinent; percentage of drug-negative urines; treatment retention	Although baseline religiosity was not significantly associated with treatment outcomes overall, higher past-year religiosity was associated with longer consecutive abstinence during treatment among participants receiving TAU + CBT4CBT-Spanish. These associations were not maintained during follow-up	USA
***Jordan et al.***,*** 2021***	Nonrandomized feasibility study	Independent study	Black adults with SUD (Mean age [SD], years = 51.9 [9.7])	40	Black	Men- 50% Women- 50%	Black church	Cocaine, alcohol, cannabis, and opioids	Delivery of CBT4CBT within a spiritual/church framework delivered by church-based health advisors (CHAs); scripture readings; prayer; praise and worship; relaxation and breathing exercises; spiritual affirmations	No control condition	Urine toxicology screens; self-reported substance use; treatment attendance/completion; treatment feasibility and retention	Participants demonstrated reductions in substance use over time based on self-report and toxicology screens. Participants reported high feasibility, adherence, and retention for the church-based CBT4CBT delivery model	USA
***Kiluk et al.***,*** 2026***	Stage 1 RCT	Independent study (Clinical trial ID: NCT03474588)	Primary Spanish-speaking adults with alcohol use disorder (Mean age (SD), years = 41.9 (12.7))	51	Hispanic	Male − 68.6%; Female − 31.4%;	Community-based outpatient treatment facilities providing services in Spanish	Alcohol	Spanish-language digital CBT4CBT-SA; telenovela format; alcohol-focused scenarios; Hispanic cultural values (*respeto*,* confianza*,* machismo*,* familismo*,* personalismo*); trauma-informed content addressing alcohol use and PTSD-related symptoms	Standard treatment (ST) compared with ST plus CBT4CBT-SA	PDA; percentage of heavy drinking days (PHDD); Alcohol Use Disorders Identification Test (AUDIT); Substance Use Calendar; Short Inventory of Problems-Revised Spanish (SIP-RS); Coping Strategies Scale (CSS)	Participants assigned to ST + CBT4CBT-SA demonstrated greater increases in percentage of days abstinent and greater reductions in heavy drinking days across the study period compared to ST alone	USA
***Papas et al.***,*** 2010***	Pilot feasibility study	Pilot study preceding Papas et al., 2020 RCT	HIV-infected outpatients with hazardous drinking (Mean Age ([SD], years = 34.2 [6.1])	27	Not reported	Men = 33.3% Women = 66.7%	AMPATH HIV outpatient clinics	Alcohol	Group paraprofessional-delivered CBT; Kiswahili-language delivery; local idioms and rural illustrations; local alcohol-related examples; gender-stratified groups; alcohol refusal skills; HIV/alcohol risk education module	No control condition	PDA; adapted TLFB; alcohol saliva tests; Clinical Institute Withdrawal Assessment for Alcohol Scale-Revised (CIWA-Ar); treatment attendance; treatment acceptability	PDA from alcohol increased from session 1 to session 6 across groups. Overall attendance was 77%. Participants reported satisfaction and acceptability with treatment and the group format	Kenya
***Papas et al.***,*** 2021***	RCT	Follow-up RCT to Papas et al., 2010 pilot study (Clinical trial ID not reported)	HIV-infected outpatients with hazardous or binge drinking (Mean age [SD], years = 38.9 [8.0]	614	Not reported	Male = 48.5% Female = 51.5%	AMPATH HIV outpatient clinics	Alcohol	Kiswahili-language group CBT; local settings and rural images; local alcohol-related examples; culturally adapted alcohol education; reduced from 12 to 6 sessions; paraprofessional delivery; gender-stratified groups	Time- and attention-controlled healthy lifestyles (HL) intervention compared with culturally adapted group CBT	PDD; drinks per drinking day (DDD); TLFB; CIWA-Ar; alcohol saliva tests; Working Alliance Inventory (WAI); Yale Adherence and Competence Scale (YACS); unprotected sex; number of sexual partners; homework adherence	Participants in the culturally adapted group CBT condition demonstrated significantly lower percentage of drinking days and drinks per drinking day compared with the HL condition across the follow-up periods. Retention and attendance were high across both conditions. Risky sexual behavior decreased over time in both conditions, with temporary short-term improvements favoring the culturally adapted group CBT participants	Kenya
***Paris et al.***,*** 2018***	RCT	Original trial (Clinical trial ID not reported)	Spanish-speaking adults meeting DSM-IV criteria for current substance abuse or dependence (Mean age [SD] = 42.9 [11.5])	92	Latino	Male = 67.4% Female = 32.6%,	Outpatient mental health and addiction treatment settings serving Latinos in New Haven, Connecticut	Alcohol, cannabis, cocaine, benzodiazepines, heroin, opiates	CBT4CBT-Spanish; telenovela format; culturally relevant experiences (e.g., immigration-related family separation); integration of Latino cultural values and concepts (e.g., *respeto*,* confianza*,* machismo*,* caballerismo*,* marianismo*,* familismo*,* fatalismo*,* sabiduría*,* personalismo*)	TAU compared with TAU plus CBT4CBT-Spanish	Days of primary substance use by week; days of any drug or alcohol use; Substance Use Calendar (Timeline Followback-style assessment); urine toxicology screens; breathalyzer screens; PDA; knowledge of cognitive and behavioral concepts	Participants assigned to TAU plus CBT4CBT-Spanish demonstrated significantly greater reductions in frequency of primary substance use compared with TAU alone, with effects maintained through 6-month follow-up. Participants assigned to TAU plus CBT4CBT-Spanish also demonstrated significantly greater increases in knowledge of cognitive and behavioral concepts	USA
***Silva et al.***,*** 2020***	Secondary analysis of RCT	Secondary analysis of Paris et al., 2018 trial (same sample; no separate ID reported)	Spanish-speaking individuals with current DSM-IV substance dependence (Mean age [SD], years = 43.0 [11.5])	83	Latino	Male- 66.3% Female- 33.7%,	Outpatient treatment centers offering services to Spanish-speaking individuals seeking treatment for substance use	Alcohol, marijuana/cannabis, cocaine, benzodiazepines, opiates, and other substances	Culturally adapted web-based CBT4CBT for Spanish-speakers; telenovela format; integration of Latino cultural values and concepts (e.g., *respeto*,* confianza*,* machismo*,* caballerismo*,* marianismo*,* familismo*,* fatalismo*,* sabiduría*,* personalismo*)	TAU compared with TAU plus CBT4CBT	Structured Clinical Interview for DSM-IV (SCID-IV); DSM-IV dependence criteria count; Substance Use Calendar; urine toxicology screens; breathalyzer screens; Addiction Severity Index (ASI); PDAfrom primary drug; PDA from all alcohol and drugs	Participants assigned to TAU + CBT4CBT demonstrated significantly greater reductions in DSM-IV dependence criteria count compared with TAU only. Lower DSM-IV dependence criteria count at end-of-treatment was associated with greater abstinence and lower legal and drug problem severity during follow-up	USA
***Webb Hooper et al.***,*** 2016***	RCT	Independent study (Clinical trial ID; NCT01811758)	African American smokers (Mean age [SD], years = 49.5 [9.1]	342	African American	Male = 56.5%; Female = 43.5%	University-based research clinic	Tobacco/nicotine	Culturally specific group CBT tailored for African Americans through race-matched clinicians and integration of culturally relevant themes (e.g., medical mistrust, spirituality, racial discrimination, family/collectivism, menthol smoking, and race-related stress)	Standard CBT for smoking cessation plus 8 weeks of nicotine patches compared with culturally specific CBT plus 8 weeks of nicotine patches	Biochemically verified 7-day point prevalence abstinence (ppa); saliva cotinine; breath carbon monoxide; Fagerström Test for Nicotine Dependence (FTND); intervention ratings; Minnesota Withdrawal Scale; intervention attendance; nicotine patch adherence	Participants in the culturally specific CBT condition were approximately twice as likely to achieve 7-day point prevalence abstinence across the study period compared with the standard CBT condition Culturally specific CBT demonstrated significantly greater abstinence at end-of-therapy and 3-month follow-up, but not at 6- or 12-month follow-up	USA

### General characteristics of included studies

Randomized controlled trials (RCTs) were the most common study design, with six studies employing this design [37, 68–72]. Three additional studies were secondary analyses of the CBT4CBT-Spanish RCT conducted by Paris et al. [70, 73–75] Three used pilot, feasibility, or nonrandomized feasibility designs [76–78]. The majority of studies were conducted in the United States, with the remaining two conducted in Kenya [37, 76]. This distribution highlights the concentration of research on ethnoracially adapted CBT for SUD within U.S. settings and the limited representation of other geographic regions.

### Sample characteristics

Sample sizes ranged from 27 to 614 participants [70, 76]. Latino/Latinx/Hispanic individuals living in the United States were the most frequently represented ethnoracial group, appearing in eight studies [68, 70–75, 78]. Target populations varied considerably across studies, including Latino adolescents with SUDs participating in culturally accommodated CBT trials [71, 72, 78]; Latino/Hispanic adults with SUDs participating in the CBT4CBT-Spanish randomized clinical trial and its subsequent secondary analyses [70, 73–75]; Spanish-speaking Hispanic adults with alcohol use disorder (AUD) [68]; HIV-infected individuals with hazardous or binge drinking in Kenya [37, 76]; African American smokers [69]; and Black adults with SUDs participating in a church-based intervention [77]. Most studies enrolled predominantly male participants. However, Jordan et al. [77]. and Papas et al.(2021) [37] included approximately equal proportions of men and women, whereas Papas et al. (2009) [76] enrolled a predominantly female sample. These findings reflect substantial heterogeneity in participant characteristics and the substance use concerns targeted across studies.

### Intervention settings and substances targeted

Most studies evaluated ethnoracially adapted CBT interventions implemented in outpatient or community-based treatment settings, including HIV clinics in both a pilot feasibility study and a subsequent RCT conducted by Papas and colleagues [37, 76], outpatient substance use and mental health treatment programs serving Latino/Hispanic adults [68, 70, 73–75], and community-based programs for Latino adolescents [71, 72, 78]. Additional interventions were delivered in a Black church setting [77] and a university-based smoking cessation clinic [69]. Alcohol was the most frequently targeted substance, appearing in 10 studies, followed by cannabis in seven studies, cocaine in six studies, opioids in four studies, benzodiazepines in two studies, and tobacco/nicotine in one study. These findings highlight the predominance of alcohol-focused interventions while also demonstrating the application of ethnoracially adapted CBT across a diverse range of substances.

### CBT-specific components adapted and control conditions

Several intervention models were identified across studies. Latino-focused interventions frequently use culturally adapted versions of Computer-Based Training for Cognitive Behavioral Therapy (CBT4CBT), a digital CBT program that teaches cognitive and behavioral coping skills through interactive multimedia modules and practice exercises [79, 80]. The CBT4CBT-Spanish RCT evaluated a culturally adapted Spanish-language version of CBT4CBT for Spanish-speaking adults with SUDs [70], and three subsequent publications reported secondary analyses of this trial [73–75]. Kiluk et al. evaluated Computer-Based Training for Cognitive Behavioral Therapy for Spanish-Speaking Adults with Alcohol Use Disorder (CBT4CBT-SA), a culturally adapted Spanish-language version developed specifically for individuals with AUD that incorporated alcohol-focused content and culturally relevant Hispanic/Latino experiences [68]. Additional intervention models included a culturally adapted smoking cessation CBT program for African American smokers [69] and a church-based CBT intervention for Black adults with SUDs [77].

Across studies, ethnoracially adapted CBT interventions incorporated a combination of surface- and deep-structure adaptations, although the type and extent of such adaptations varied across intervention models and target populations. Surface-structure adaptations most commonly involved language translation and culturally tailored delivery formats. For example, the Kenyan interventions delivered CBT in Kiswahili and incorporated locally relevant illustrations, alcohol-related examples, and rural imagery [37, 76]. Similarly, culturally adapted Spanish-language interventions incorporated Spanish-language delivery, telenovela-style narratives, culturally familiar characters, and multimedia content designed to enhance accessibility and engagement among Hispanic/Latino participants [68, 70]. Deep-structure adaptations integrated culturally salient values, identities, experiences, and belief systems into the core components of CBT. Among Latino/Hispanic adults, CBT4CBT adaptations incorporated cultural constructs such as *familismo*, *personalismo*, *respeto*, *confianza*, *machismo*, *marianismo*,* caballerismo*, f*atalismo*, and *sabiduría* into treatment content and scenarios [68, 70]. Burrow-Sánchez and colleagues adapted CBT for Latino adolescents by incorporating ethnic identity development, acculturation experiences, discrimination, ethnic identity matching, and increased family involvement [71, 72, 78] Jordan et al. integrated spirituality into CBT delivery through scripture readings, prayer, praise and worship, spiritual affirmations, and a church-based health advisor [77]. Webb Hooper et al. addressed deep-structure cultural factors relevant to African American smokers, such as experiences of racism, medical mistrust, racial stress, spirituality, collectivism, and menthol cigarette use among African American smokers [69]. Finally, the Kenyan interventions incorporated HIV-specific content, alcohol-refusal skills, gender-stratified groups, and locally relevant drinking norms to enhance cultural relevance in the Kenyan context [37, 76].

Control conditions varied substantially across studies. Several trials compared culturally adapted CBT with treatment as usual (TAU) [70, 73–75], standard treatment delivered in community-based outpatient settings [68], or standard manualized CBT without cultural adaptation [69, 71, 72, 78]. In studies using TAU, participants typically received standard outpatient SUD treatment services, including individual or group counseling, pharmacotherapy when indicated, and case management [70]. In these studies, culturally adapted CBT was delivered in addition to TAU rather than as a stand-alone intervention. Papas et al. compared culturally adapted group CBT with a time- and attention-matched Healthy Lifestyles (HL) intervention, an active control condition designed to match the CBT intervention in duration, group format, and participant-provider contact while focusing on general health promotion rather than alcohol use reduction [37]. Two studies did not include a control condition and were primarily designed to evaluate feasibility, acceptability, implementation, and preliminary intervention outcomes [76, 77]. Notably, only a small number of studies directly compared culturally adapted CBT with standard CBT without cultural adaptation, limiting the ability to isolate the specific contribution of cultural adaptation to treatment outcomes [69, 71, 72, 78].

### Outcome measures and findings

The Timeline Followback (TLFB) interview, or a comparable calendar-based substance use assessment, was the most frequently utilized outcome measure, appearing in multiple studies [68, 70–75, 78]. The TLFB provides a retrospective quantitative assessment of alcohol and other substance use over a specified period [81]. Several studies also assessed factors associated with treatment response, including coping skills assessed through the Drug Risk Response Test (DRRT) [75], religiosity assessed through the Religious Background and Behavior Questionnaire [73], and measures of acculturation and ethnic identity, such as the Acculturation Rating Scale for Mexican Americans-II (ARSMA-II) and the Multigroup Ethnic Identity Measure (MEIM) [78]. Several studies further incorporated biological verification methods, including urine toxicology screens, saliva testing, and breathalyzer assessments, to corroborate self-reported substance use outcomes. Primary outcomes assessed across studies included substance use reduction, abstinence, treatment retention, coping skill acquisition, treatment satisfaction, therapeutic alliance, and intervention feasibility.

Most studies reported reductions in substance use following participation in culturally adapted CBT interventions. Evidence from randomized controlled trials comparing culturally adapted CBT with TAU, standard treatment, active control interventions, or standard CBT generally supported favorable substance use outcomes. Paris et al. found that participants receiving culturally adapted CBT4CBT-Spanish in addition to TAU demonstrated greater reductions in substance use than those receiving TAU alone [70]. Secondary analyses of this trial further examined factors associated with treatment outcomes. Benitez et al. found that higher-quality coping skills were associated with lower substance use and that participants receiving CBT4CBT-Spanish demonstrated greater improvements in coping skills during treatment than those receiving TAU alone [75]. Jaramillo et al. reported that higher levels of religiosity were associated with longer periods of abstinence during treatment among participants receiving CBT4CBT-Spanish, although these associations were not maintained at follow-up [73]. Silva et al. found that participants receiving CBT4CBT-Spanish demonstrated greater reductions in DSM-IV dependence severity, and lower dependence severity at the end of treatment was associated with greater abstinence and lower drug- and legal-related problem severity during follow-up [74]. Similarly, Kiluk et al. reported improved alcohol-related outcomes among participants receiving culturally adapted CBT4CBT-SA in addition to standard treatment [68]. Papas et al. found lower drinking frequency and intensity among participants receiving culturally adapted group CBT compared with an active HL intervention that matched the CBT condition in duration, format, and participant-provider contact [37].

Among the studies that directly compared culturally adapted CBT with standard CBT without cultural adaptation [69, 71, 72, 78], the adolescent-focused trials found that both culturally adapted and standard CBT produced significant reductions in substance use [71, 72, 78]. However, the 12-month follow-up study reported significantly lower substance use among adolescents receiving culturally accommodated CBT compared with standard CBT, with parental *familismo* significantly moderating treatment outcomes [72]. Across these studies, ethnic identity and *familismo* were associated with differential treatment response across intervention conditions [71, 72, 78]. In contrast, Webb Hooper et al. reported higher smoking abstinence rates among participants receiving culturally specific CBT compared with standard CBT [69].

Findings related to intervention acceptability and engagement were primarily reported in studies examining Spanish-language adaptations of CBT4CBT [68, 70, 73–75]. These studies reported favorable findings related to treatment engagement, retention, acceptability, and participant satisfaction. Participants described the Spanish-language, telenovela-style modules as culturally relevant, understandable, and engaging [68, 70].

## Discussion

This scoping review synthesized the literature on ethnoracially adapted cognitive behavioral therapy (CBT) interventions for substance use disorders (SUDs) to examine how cultural adaptations have been conceptualized, operationalized, and evaluated across diverse populations. Overall, the findings indicate that ethnoracial adaptations most commonly combined surface-structure modifications, such as language translation and culturally tailored delivery formats, with deep-structure modifications that integrated culturally salient values, identities, and belief systems into core CBT components. Across studies, ethnoracially adapted CBT interventions were generally associated with favorable substance use outcomes. Additionally, the evidence base was concentrated largely among Latino/Hispanic populations in the United States, with comparatively limited representation of other ethnoracial groups and geographic regions, thereby constraining the generalizability of current findings.

A key finding of this review was the considerable heterogeneity in how cultural adaptations were operationalized across studies. Adaptations ranged from surface-structure modifications to deep-structure modifications, and many interventions combined both approaches [37, 68, 70–73]. This finding is consistent with prior literature suggesting that culturally responsive interventions may be more relevant, acceptable, and effective when they employ both approaches [30, 33, 42, 82] Such adaptations may enhance intervention relevance, engagement, and acceptability by addressing limitations of standard CBT approaches that may not fully account for culturally specific experiences, values, and contextual stressors [33, 58, 83].

Another notable finding of this review was the substantial variation in comparator conditions used to evaluate culturally adapted CBT interventions, which has important implications for interpreting the evidence base. Although the reviewed studies generally reported favorable outcomes following culturally adapted CBT interventions, few directly compared culturally adapted CBT with its standard CBT equivalent. Consequently, it remains difficult to determine the extent to which observed treatment effects are attributable to the cultural adaptations themselves rather than to CBT more broadly. Nevertheless, the small number of direct comparison studies identified in this review provides preliminary evidence that cultural adaptations may influence treatment response under certain conditions. Notably, several studies included in this review identified ethnic identity, *familismo*, and religiosity as factors associated with differential treatment response, suggesting that culturally salient characteristics may help explain for whom and under what circumstances culturally adapted interventions are most beneficial [71–73, 78]. This observation is consistent with prior research demonstrating that cultural factors such as ethnic identity, family processes, and religiosity are associated with substance use behaviors and treatment-related outcomes among ethnoracially diverse populations [42, 71, 72, 84–86].

Despite promising findings, implementation outcomes were inconsistently reported across the included studies. Although several studies reported favorable findings related to participant engagement, retention, satisfaction, feasibility, and acceptability, these outcomes were not assessed systematically or consistently across interventions [68, 70, 73, 75–77]. While most studies described the cultural elements incorporated into the intervention, few provided substantial details regarding how adaptations were developed, the extent of community involvement, or the use of formal adaptation frameworks [37, 68, 69, 71, 72, 77]. These reporting gaps constrain replication efforts and limit understanding of which adaptation and implementation strategies are most strongly associated with intervention outcomes [30, 83, 87, 88]. Long-term follow-up data were also limited, with most studies assessing outcomes at post-treatment or within relatively short follow-up periods. Notably, one of the few studies reporting 12-month outcomes found significantly lower substance use among adolescents receiving culturally accommodated CBT compared with standard CBT, whereas no significant differences were observed at earlier follow-up assessments [72]. This finding suggests that potential effects of cultural adaptation may not be fully apparent during the immediate post-treatment period and underscores the importance of incorporating longer-term follow-up assessments in future research [72, 87].

Ethnoracial, geographic, and economic representation revealed important gaps in the current evidence base. Most studies were conducted in the United States and focused on Latino/Hispanic populations, with only two studies conducted in Kenya [37, 76]. Consequently, other ethnoracial groups and geographic regions, particularly populations living in LMICs, were substantially underrepresented. Although the current literature on ethnoracially adapted CBT for Latino/Hispanic populations provides important insights into the cultural adaptation of evidence-based substance use interventions [42], there is still a need to expand adaptation research to other ethnoracial populations and cultural contexts [42, 63].

### Implications and future directions

Findings from this review suggest that ethnoracially adapted CBT is a promising approach for addressing SUDs across diverse populations. Across studies, adaptations frequently extended beyond language translation to incorporate culturally relevant values, beliefs, experiences, and social contexts into treatment content and delivery. Clinicians and intervention developers should therefore consider integrating cultural constructs such as *familismo*, spirituality, and ethnic identity, as well as experiences relevant to ethnoracial groups, including discrimination, racial stress, acculturation, and medical mistrust, when adapting CBT for specific populations. The review also highlights the potential value of community engagement during the adaptation process. Several studies incorporated stakeholder input, formative research, or culturally informed adaptation procedures [37, 71, 72], suggesting that collaboration with members of the target population may help enhance the cultural relevance and acceptability of adapted interventions [89]. Future adaptation efforts should more consistently engage community stakeholders throughout intervention development, refinement, and implementation, and examine the extent to which community involvement influences intervention acceptability, feasibility, engagement, and treatment outcomes [89–91].

Several priorities for future research also emerged. First, studies should expand beyond predominantly U.S.-based Latino/Hispanic populations to include a broader range of ethnoracial groups and geographic regions, particularly LMICs, where culturally adapted SUD interventions remain understudied, yet the SUD burden is substantial [9–11]. Second, future research should move beyond demonstrating effectiveness to identifying the specific mechanisms through which cultural adaptations influence treatment outcomes. The findings of this review suggest that factors such as ethnic identity, *familismo*, and religiosity may help explain variation in treatment response, highlighting the importance of examining culturally relevant moderators and mechanisms of change. At the same time, some culturally salient constructs, such as spirituality and religiosity, may be relevant across multiple ethnoracial groups, although their specific meanings, expressions, and practices may vary both between and within populations [42]. Future research should therefore move beyond simply incorporating cultural elements into interventions and instead more rigorously examine how these elements influence treatment engagement, responsiveness, and substance use outcomes. Finally, greater attention should be devoted to implementation outcomes, including fidelity, acceptability, feasibility, sustainability, cost-effectiveness, and longer-term treatment outcomes. More comprehensive evaluation of these domains will improve understanding of how ethnoracially adapted CBT interventions are implemented, sustained, and scaled across diverse settings. Such efforts may also help clarify which adaptation and implementation strategies are associated with improved engagement, retention, and substance use outcomes, thereby supporting the broader dissemination of culturally responsive CBT interventions.

### Limitations

This review should be interpreted in light of the following limitations. First, as a scoping review, it prioritized breadth over depth and did not assess the methodological quality or risk of bias in the included studies, thereby limiting the evaluation of the overall strength of the evidence. Second, inclusion was restricted to peer-reviewed, English-language publications, potentially excluding relevant studies from non-English-speaking regions or grey literature sources. Third, variation in the terminology used to describe cultural adaptation may have led to the omission of some relevant studies despite the comprehensive search strategy employed. Fourth, substantial heterogeneity in intervention characteristics, cultural adaptation strategies, comparator conditions, and outcome measures limited direct comparisons across studies. In particular, variability in comparator conditions made it difficult to determine whether observed outcomes were attributable to the cultural adaptations themselves, the underlying CBT intervention, or other aspects of treatment delivery. Fifth, many studies provided limited detail regarding adaptation procedures, community involvement, and implementation processes, constraining replication and limiting understanding of which adaptation strategies were most strongly associated with treatment engagement and substance use outcomes. Sixth, the review focused exclusively on ethnoracial adaptations of CBT and on interventions in which CBT was the primary treatment modality, potentially excluding relevant multimodal interventions or adaptations targeting other dimensions of identity and lived experience. Finally, the evidence base was largely concentrated among Latino/Hispanic populations in the United States, limiting the generalizability of the findings to other ethnoracial groups, geographic regions, and LMICs.

## Conclusions

This scoping review contributes to the growing literature on ethnoracially adapted CBT interventions for SUDs. The findings highlight the diverse ways in which cultural adaptations have been operationalized, ranging from language-based modifications to the integration of culturally relevant themes into core CBT components. Overall, ethnoracially adapted CBT interventions were generally associated with favorable substance use outcomes; however, limited direct comparisons with standard CBT constrain conclusions regarding the extent of the unique contribution of cultural adaptation to treatment effectiveness. Important gaps remain in implementation reporting, geographic representation, and understanding of the mechanisms by which cultural adaptations influence long-term treatment outcomes. Future research should prioritize rigorous evaluation of adaptation-specific effects, longer-term outcomes, and broader ethnoracial and geographic representation. Ultimately, strengthening this evidence base will be essential for guiding the development, implementation, and scale-up of culturally responsive CBT interventions and advancing more equitable substance use treatment systems globally.

## Data Availability

No datasets were generated or analysed during the current study.
